# Nutritional and Phytochemical Composition and Antioxidant Activity of Edible Stems of Smooth Cordgrass (*Spartina alterniflora*)

**DOI:** 10.3390/foods13193150

**Published:** 2024-10-02

**Authors:** Yijuan Han, Huiquan Chen, Meiling Lai, Zhongyuan Lin, Yongji Huang, Weiqi Tang, Yanbing Zhu, Yange Zhang, Zonghua Wang, Hui Ni, Xiaofeng Chen, Songbiao Chen

**Affiliations:** 1 Fuzhou Institute of Oceanography, Minjiang University, Fuzhou 350108, China; 2 Fujian Provincial Universities Engineering Research Center of Marine Biology and Drugs, College of Geography and Oceanography, Minjiang University, Fuzhou 350108, China; 3 Fujian University Key Laboratory for Plant-Microbe Interaction, College of Plant Protection, Fujian Agriculture and Forestry University, Fuzhou 350002, China; 4 College of Food and Biological Engineering, Jimei University, Xiamen 361021, China

**Keywords:** smooth cordgrass, *Spartina alterniflora*, edible stems, nutritional composition, antioxidant activity

## Abstract

Smooth cordgrass (*Spartina alterniflora*) is a native salt marsh plant along the Atlantic coast but has become an invasive species in coastal regions in China, as well as other areas. Utilizing it for resources has become a control measure in reducing the spread of *S. alterniflora*. This study assesses the nutritional and phytochemical properties of the edible stems of *S. alterniflora* collected from three locations in Fujian province, China. The tender stems of *S. alterniflora* exhibit a rich nutritional profile, with high levels of protein, carbohydrates, and fats, and significant amounts of essential vitamins, minerals, and antioxidants, indicating their potential as a nutritious addition to the diet or forage. In addition, the levels of potential contaminants, including nitrate, nitrite, cadmium, lead, and chromium, are below the established safe thresholds for consumption. Our results provide valuable information for the sustainable utilization of *S. alterniflora* resources and will contribute to the integrated control of *S. alterniflora.*

## 1. Introduction

Smooth cordgrass (*Spartina alterniflora*) is a species of cordgrass within the family *Poaceae* and is native to the intertidal zones along the Atlantic and Gulf coastal areas of North America [1]. *S. alterniflora* plays a crucial ecological role in native coastal ecosystems by stabilizing coastal sediments, acting as a natural barrier against storm surges and tidal forces, and helping mitigate the impact of extreme weather events on coastal communities [2,3,4,5]. The dense stands of *S. alterniflora* also serve as vital habitats and nurseries for diverse wildlife, enhancing biodiversity in marine and estuarine areas [3,6,7]. Moreover, the root system of *S. alterniflora* filters and traps pollutants, thereby improving water quality in estuarine and coastal regions [3,8]. *S. alterniflora* can also have benefits in terms of carbon sequestration and cyclic nutrient dynamics within the ecosystem [9,10].

*S. alterniflora* was introduced to China in 1979 with the aim of mitigating tidal impacts and promoting the accretion of coastal wetlands [11,12]. However, its aggressive growth and rapid spread have led to competitive dominance over native vegetation, resulting in dense monocultures that displace other plant species and diminish native plant diversity. This has negatively affected native fauna that rely on those native plants for food and habitats [13,14,15,16,17]. The absence of natural predators in China has allowed *S. alterniflora* to proliferate uncontrolled, leading to its continuous dominance in new environments. Along the coasts of Jiangsu, Shanghai, Zhejiang, and Fujian provinces, *S. alterniflora* has covered over 60% of coastal areas, making it the most concentrated region for this species in the country [15,18,19,20]. The invasiveness of *S. alterniflora* poses challenges for restoration efforts aiming to reintroduce native vegetation [17,21,22]. Overall, the invasion by *S. alterniflora* of China’s coastal wetlands has caused serious negative effects on ecosystem health, biodiversity conservation, and sustainable coastal management.

Over the past several decades, both the national and local governments have implemented control measures to reduce the spread of *S. alterniflora* in China. Various strategies have been proposed to eradicate *S. alterniflora* and restore the native ecosystem, including uprooting or cutting down the grass, using chemical herbicides that target *S. alterniflora* specifically, introducing natural predators or pathogens, and replanting native plant species after clearing *S. alterniflora* [23,24,25,26,27]; however, the threat posed by *S. alterniflora* to China’s coastal ecosystem remains unresolved. On the other hand, several strategies have also been proposed to utilize *S. alterniflora* for resources, such as forage for aquaculture [28,29] and livestock [12,30], culture medium for mushrooms [30,31], and feedstock for bioenergy [32].

Unconventional food plants have emerged as a promising source in the food chain in recent years due to their outstanding amounts of proteins, minerals, and bioactive compounds [33]. These plants offer a way to diversify the food or pharmaceutical supply but with a lower impact on the environment. During our investigation into controlling *S. alterniflora* in the Ningde area of Fujian Province from 2000 to 2023, we found that local residents had been using the tender stems of *S. alterniflora* as a vegetable for decades.

In the present study, we explored the nutritional value and bioactive compounds of the tender stems of *S. alterniflora* and evaluated its food safety. The tender stems of *S. alterniflora* were rich in multiple nutrients, including protein, carbohydrates, fat, dietary fiber, mineral elements, vitamins, and amino acids. The tender stems also contained medium level of antioxidant substances with medium levels of antioxidant scavenging activities. The contents of nitrate, nitrite, cadmium, lead, and chromium in tender shoots all met the limits set by food safety regulations. Our results provide valuable information for the utilization of resources from *S. alterniflora* as an alternative food for humans and domestic animals.

## 2. Materials and Methods

### 2.1. Materials

Newly growing tillers of *S. alterniflora* were harvested during March and April in 2022 from three tidal flat areas (Xiapu, Lianjian, and Quanzhou) in Fujian, China, with coordinates 119°52′23.88″ E, 26°40′31.91″ N; 119°40′33.51″ E, 26°16′6.40″ N; and 118°39′29.63″ E, 24°54′24.15″ N, respectively. *S. alterniflora* shoots in each area were randomly harvested from 60 to 100 plants, with each plant spaced approximately 10 m apart to minimize environment variability. After cleaning and discarding the inedible parts (leaves, leaf sheaths, etc.), about 600 g tender stems were prepared. Each measurement was conducted by using at least triplicate samples. For each sampling location, 200 g of *S. alterniflora* tender stems were used as one repeat for multiple extracts or measurements.

### 2.2. Nutritional Composition Analysis

Moisture content was determined by measuring the weight loss after oven-drying to constant weight at 103 °C. Ash content was determined at 550 °C according to ASTM standard D1102-84 [34]. Crude protein content was measured by the AOAC 981.10 Kjeldahl method [35], with a nitrogen conversion factor of 6.25 [36].

Crude fiber content was analyzed by the gravimetric method (AOAC method 991.43) as described by Ferjančič et al. [37]. Total carbohydrate content was determined by the phenol–sulfuric acid method as detailed by Nielsen and Carpenter [38]. Briefly, 0.2 g of plant tissue was placed into a 50 mL boiling tube and suspended with 10 mL distilled water. About 3 mL of sulfuric acid and 0.6 mL of 80% phenol was added to the suspension. The mixtures were boiled with water for 3 h and were filtrated. The filtrated solution was filed with water to a final volume of 50 mL and tested for absorbance at 490 nm.

Reducing sugars were quantified by the dinitrosalicylic acid (DNS) method as described by Teixeira et al. [39]. About 0.5 g of plant tissue was suspended in 10 mL distilled water and incubated at 50 °C for 20 min to extract the reducing sugars. The supernatant was mixed with DNS reagent and boiled in water for 5 min. The solution was tested for absorbance at 540 nm. Crude fat was extracted by using a Soxhlet apparatus with petroleum ether and was determined following the method as described by Nielsen and Carpenter [40].

Total soluble amino acids were measured by the ninhydrin method as described by Sun et al. [41]. Individual amino acid composition was analyzed by HPLC-MS/MS (Agilent 1260 and AB 4000, Santa Clara, CA, USA). HPLC was set as follows: chromatographic column, Information-HILICZ (2.7 µm, 3.0 × 100); column temperature, 35 °C; flow phase, 75% acetonitrile in water, 0.1 mol/L ferric acetate; flow rate, 0.3 mL/min; and input volume, 1 µL. MS was set as follow: ionization mode, ESI positive ion mode; scan type, MRM; curtain air, 15 psi; spray voltage, +4000 V; atomizing gas pressure, 65 psi; auxiliary air pressure, 70 psi; and atomization temperature, 400 °C. The ratios of essential amino acids, bitter amino acids, umami amino acids, and sweet amino acids were calculated based on their proportion in the total amino acid content [33]. The contents of vitamins B1, B2, B3, B6, and vitamin C were determined by using HLPC (Agilent, 1260 HPLC).

The energy of tender stems of *S. alterniflora* was calculated via total protein content × 4 + total fat content × 9 + carbohydrate content × 4, and presented in kcal/100 g.

### 2.3. Determination of Macro Elements, Trace Elements and Heavy Metals

The contents of five macro elements—sodium, potassium, calcium, magnesium, and phosphorus—were determined using atomic emission spectroscopy (Agilent 7101 ICP-OES). The contents of nine trace elements and heavy metals—iron, zinc, manganese, copper, boron, selenium, cadmium, lead, and chromium—were quantified by using Agilent 7900 ICP-MS.

### 2.4. Determination of Contents of Total Phenols, Total Flavonoids, Total Alkaloids, Total Nitrite, and Total Nitrate

The total phenol content was determined using the Folin–Ciocalteu phenol colorimetric assay as described by Attard [42]. Gallic acid was used as a standard to prepare a standard curve for total phenols, with absorbance readings taken at 510 nm. The total flavonoid content was determined by the sodium nitrite–aluminum nitrate colorimetric assay as described by Ma et al. [43], employing rutin as the standard to generate the standard curve, and absorbance was measured at 765 nm. The total alkaloid content was quantified by the Folinol colorimetric method as described by Ajanal et al. [44]. Determination of nitrite and nitrate contents was carried with the N-(1-Naphthyl) ethylene diamine method as described by Prasad and Chetty [45].

### 2.5. Determination of Antioxidative Activities

ABTS^+^ radical scavenging activity was measured using an ABTS assay kit (ABTS-2-D) following the manufacturer’s instructions (Cominbio, Suzhou, China). Absorbance measurements for each sample were taken at 734 nm, and the ABTS radical scavenging rate was calculated as μmol of trolox equivalents per 100 g of *S. alterniflora* tender stems (µmol trolox/100 g).

DPPH (2,2′-diphenyl-1-picrylhydrazyl) radical scavenging activity was measured with a DPPH assay kit (DPPH-2-D) following the manufacturer’s instructions (Cominbio, Suzhou, China). Absorbance readings for each sample were recorded at 519 nm using a microplate reader (Read max 300, Shanghai Flash, Shanghai, China). The DPPH radical scavenging rate was presented as µmol of trolox equivalents per 100 g of *S. alterniflora* tender stems (µM trolox/100 g).

Ferric-reducing antioxidant power (FRAP) was measured using an FRAP assay kit (FRAP-2-G) following the manufacturer’s instructions (Cominbio, Suzhou, China). The reduction of Fe^3+^-TPTZ to Fe^2+^-TPTZ was measured at 593 nm and expressed as µmol of trolox equivalents per 100 g of *S. alterniflora* tender stems (µmol trolox/100 g).

Hydroxyl radical scavenging activity was measured using a hydroxyl radical scavenging assay kit (QZQ-2-G) following the manufacturer’s instructions (Cominbio, Suzhou, China). The reaction mixture was incubated for 1 h at 37 °C, and absorbance was measured at 510 nm. The hydroxyl radical scavenging rate was calculated using the following formula: (Absorbance control − Absorbance sample) ÷ (Absorbance control − Absorbance mock) × 100%.

### 2.6. Statistical Analyses

All assays were performed in triplicate, with values presented as mean ± standard deviation (SD). Differences between means were analyzed by using the Tukey test following a one-way analysis of variance (ANOVA) with GraphPad Prism software version 8. Statistical significance was determined at a threshold of *p* < 0.05.

## 3. Results and Discussions

### 3.1. The Nutritional Composition of Tender Stems of S. alterniflora

**Figure 1:**
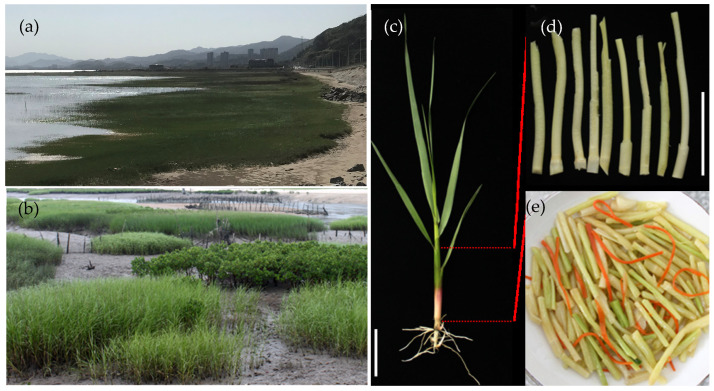
Views of smooth cord grass (*S. alterniflora*) in the coastal areas of Fujian. Smooth cord grass in Lianjiang county, Fuzhou, in early April 2022 (**a**) and in Quanzhou city in May 2022 (**b**); (**c**), seedling of *S. alterniflora* sampled from Xiapu county, Ningde; (**d**) tender and edible stems of *S. alterniflora* harvested from Xiapu; (**e**) the *S. alterniflora* cuisine served in local restaurants in Xiapu county. The scales in (**c**,**d**) are presented for 5 cm and 1 cm length, respectively.

**Figure 2:**
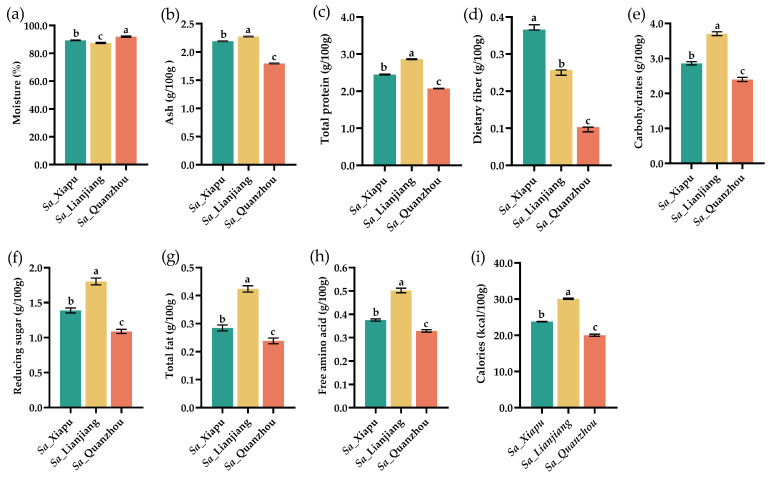
Proximal compositions of *S. alterniflora* tender stems. The amounts of moisture (**a**), ash (**b**), total protein (**c**), dietary fiber (**d**), carbohydrates (**e**), reducing sugar (**f**), total fat (**g**), free amino acid (**h**), and calories (**i**) were evaluated in *S. alterniflora* tender stems from Xiapu (indicated as *Sa*_Xiapu), Lianjiang (indicated as *Sa*_Lianjiang), and Quanzhou (indicated *Sa*_Quanzhou) areas, respectively. Different letters (a–c) above the columns indicate statistical differences (*p* < 0.05).

Over the past decades, local residents in Xiapu, China, have exploited the tender stems of *S. alterniflora* as a unique vegetable (Figure 1) during the spring season. The newly growing tillers of *S. alterniflora* were harvested from clean coastal areas (Figure 1a–c). Subsequently, after cleaning and stripping the leaves, sheaths and roots, the tender stems were used as edible parts (Figure 1d,e). These tender stems have been called “Sea Bamboo Shoots” in Xiapu. In this study, we first investigated the nutritional composition of the tender stems of *S. alterniflora* collected from three coastal locations in Fujian, China, including Xiapu, Lianjiang, and Quanzhou (designated as *Sa*_Xiapu, *Sa*_Lianjiang, and *Sa*_Quanzhou, respectively). The proximate composition of the *S. alterniflora* tender stems collected from three locations is detailed in Figure 2.

The fresh *S. alterniflora* tender stems contained moisture contents ranging from 87.45% to 92.01% (Figure 2a), ash contents between 1.80 and 2.27 g/100 g (Figure 2b), crude protein contents of 2.07–2.86 g/100 g (Figure 2c), dietary fiber contents of 0.10–0.37 g/100 g (Figure 2d), total carbohydrate contents of 2.40–3.71 g/100 g (Figure 2e), reducing sugar contents of 1.09–1.80 g/100 g (Figure 2f), crude fat contents of 0.24–0.42 g/100 g (Figure 2g), total free amino acid contents of 0.33–0.50 g/100 g (Figure 2h), and calories of 20.03–30.09 kcal/100 g (Figure 2i).

The study also revealed variations in the nutritional compositions of the *S. alterniflora* tender stems harvested from different locations. The *Sa*_Xiapu and *Sa*_Lianjiang tender stems displayed higher levels of most of the tested characteristics compared to the *Sa*_Quanzhou tender stems, with the exception of moisture content, which could be associated with variations in climate, weather, and soil nutrients occuring along the latitudinal gradients.

The nutritional composition of the *S. alterniflora* tender stems is comparable with that of some other stem vegetables. For instance, 100 g of raw bamboo shoots contains approximately 0.9 g of ash, 2.6 g of protein, 2.2 g of fiber, 5.2 g of carbohydrate, and 0.3 g of fat [46]. Fresh raw asparagus (*A. officinalis*) contains about 2.2% crude protein, 0.12% crude lipid, 2.1% crude fiber, and 1.9% carbohydrates and supplies 20 kcal of energy [47,48]. Similarly, wild rice swollen culm (*Zizania latifolia*), known as “Jiao Bai” in China, which is an aquatic vegetable renowned in East Asia, contains 1.15–1.35 g/100 g of crude protein, 2.87–3.90 g/100 g of reducing sugar, 2.43 g/100 g of carbohydrates, 2.26 g/100 g of fat, and 4.2 g/100 g of fiber [49,50,51]. Overall, the *S. alterniflora* tender stems exhibited remarkable nutritional profiles, surpassing those of some traditional vegetables like asparagus and wild rice swollen culm. The high levels of total protein in *S. alterniflora* tender stems could due to the presence of adequate nitrogen nutrients in tidal flat areas [52], which is essential for robust plant growth and higher protein content in vegetables. Along with an abundance of carbohydrates and fats, *S. alterniflora* tender stem could serve as a valuable nutrient supplement, highlighting their potential as vegetable foods.

### 3.2. Amino Acid Profiles

The contents of nine essential amino acids (histidine, isoleucine, leucine, lysine, methionine, phenylalanine, threonine, tryptophan, and valine) and eleven non-essential amino acids (alanine, arginine, asparagine, aspartic acid, cysteine, glutamic acid, glutamine, glycine, proline, serine, and tyrosine) in the *S. alterniflora* tender stems were measured and are listed in Table 1. Among the amino acids whose content was analyzed, asparagine was found to be the most abundant (ranging from 61.4 to 201.08 mg/100 g), while methionine was the least abundant (ranging from 0.66 to 1.14 mg/100 g), suggesting it could be the limiting amino acid in *S. alterniflora.* The levels of essential and non-essential amino acids in the *S. alterniflora* tender stems varied depending on the location where they were harvested. For example, the histidine content ranged from 2.8 mg/100 g (*Sa*_Lianjiang) to 34 mg/100 g (*Sa*_Quanzhou). Overall, the *Sa*_Quanzhou tender stems had a higher total essential amino acid (TEAA) level (92.36 mg/100 g) compared to the *Sa*_Xiapu (69.61 mg/100 g) and *Sa*_Lianjiang (61.79 mg/100 g) samples. In contrast, the total non-essential amino acid (TNEAA) level in the *Sa*_Lianjiang tender stems (356.95 mg/100 g) was at least 24% higher than that in the *Sa*_Xiapu (287.04 mg/100 g) and *Sa*_Quanzhou (192.71 mg/100 g) samples.

The TEAA concentration in the tender stems of *S. alterniflora*, averaging 74.59 mg/100 g, was significantly higher than that found in the swollen culm of *Z. latifolia* (38.7 mg/100 g) [53] and in wild asparagus (21.47 mg/100 g) [54], indicating that *S. alterniflora* tender stems could provide a much higher level of essential amino acids. In contrast, the content of total non-essential amino acid (TNEAA) in the tender stems of *S. alterniflora*, averaging 278.9 mg/100 g, was lower than that in the swollen culms of *Z. latifolia* (349.01 mg/100 g) [53] and in wild asparagus (336.41 mg/100 g) [54]. The total amino acid (TAA) in the tender stems of *S. alterniflora*, averaging 353.48 mg/100 g, was comparable to that in asparagus (357.88 mg/100 g) but lower than that in *Z. latifolia* swollen culms (407.98 mg/100 g) [53,54]. Although the *S. alterniflora* stems may have a lower content of non-essential amino acids compared to other vegetables, their overall amino acid content could position them as a viable option for fulfilling essential amino acid requirements in diets or supplement formulations.

The *Sa*_Xiapu tender stems had the highest content of total umami amino acids (TUAA, composed of glutamic acid and aspartic acid), at 71.21 mg/100 g, followed by the *Sa*_Lianjiang sample at 59.28 mg/100 g and the *Sa*_Quanzhou sample at 33.92 mg/100 g. In terms of the content of total bitter amino acid (TBAA, including proline, valine, leucine, phenylalanine, and tryptophan), the *Sa*_Quanzhou tender stems recorded the highest level at 90.55 mg/100 g, followed by *Sa*_Xiapu at 82.71 mg/100 g and *Sa*_Lianjiang at 74.21 mg/100 g. As for the content of total amino acid (TAA), the *Sa*_Lianjiang tender stems exhibited the highest level at 418.73 mg/100 g, followed by *Sa*_Xiapu at 356.65 mg/100 g and *Sa*_Quanzhou at 285.07 mg/100 g, which could be a reason for the common consumption of this species in northen Fujian. These variations in amino acid levels may be influenced by factors such as soil type, climate, and other environmental conditions.

### 3.3. Vitamins

**Figure 3:**
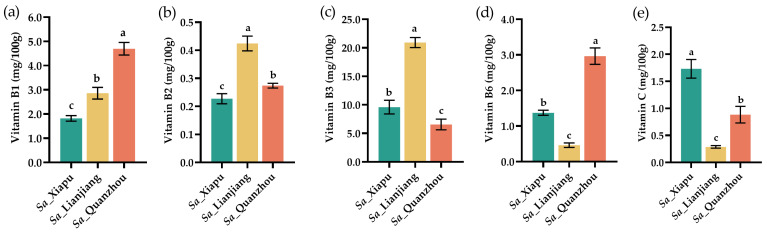
Contents of vitamins in of *S. alterniflora* tender stems (mg/100 g, fresh weight). The amounts of vitamin B1 (**a**), vitamin B2 (**b**), vitamin B3 (**c**), vitamin B6 (**d**), and vitamin C (**e**) were evaluated in *S. alterniflora* tender stems from the Xiapu (indicated as *Sa*_Xiapu), Lianjiang (*Sa*_Lianjiang), and Quanzhou (*Sa*_Quanzhou) areas. The error bars in each column were calculated based on standard deviation (SD) in three replicates. Different letters (a–c) above the columns indicate statistical differences (*p* < 0.05).

The contents of vitamins, including vitamin B1 (thiamin), B2 (riboflavin), B3 (niacin), B6 (pyridoxine), and C (ascorbic acid), in the *S. alterniflora* tender stems were measured and are depicted in Figure 3. The *S. alterniflora* tender stems accumulate abundant levels of vitamin B1, B2, B3, and B6. Among the tested samples, the *Sa*_Quanzhou tender stems exhibited the highest levels of vitamin B1, at 4.69 mg/100 g (Figure 3a), compared to the *Sa*_Lianjiang (2.86 mg/100 g) and the *Sa*_Xiapu (2.85 mg/100 g) samples, which is notably higher than the *Sa*_Lianjiang (2.86 mg/100 g) and *Sa*_Xiapu (2.85 mg/100 g) samples and substantially greater than the levels found in *Z. latifolia* swollen culms (0.02 mg/100 g) [51], leeks (0.06 mg/100 g) [55], asparagus (0.14 mg/100 g) [56], and bamboo shoots (0.15 mg/100 g) [46].

*Sa*_Lianjiang tender stems displayed the highest vitamin B2 content, at 424.53 mg/100 g (Figure 3b), compared to the *Sa*_Quanzhou (273.79 mg/100 g) and the *Sa*_Xiapu (227.43 mg/100 g) samples, with these contents being significantly greater than those found in leeks (0.03 mg/100 g) [55], bamboo shoots (0.07 mg/100 g) [46], and asparagus (0.14 mg/100 g) [56].

Similarly, regarding vitamin B3, the *Sa*_Lianjiang tender stems had the highest levels at 20.93 mg/100 g (Figure 3c), followed by the *Sa*_Xiapu (9.6 mg/100 g) and the *Sa*_Quanzhou (6.55 mg/100 g) samples. These levels are greater than those in leeks (0.4 mg/100 g) [55], bamboo shoots (0.6 mg/100 g) [46], and asparagus (0.98 mg/100 g) [56].

Regarding vitamin B6, the *Sa*_Quanzhou tender stems exhibited the highest content (2.97 mg/100 g), followed by the *Sa*_Xiapu (1.37 mg/100 g) and the *Sa*_Lianjiang (0.46 mg/100 g) samples (Figure 3d). This is notably higher than the levels in celery (0.05 mg/100 g) [57], asparagus (0.09 mg/100 g) [56], leeks (0.23 mg/100 g) [55], and bamboo shoots (0.24 mg/100 g) [45], indicating that *S. alterniflora* tender stems could be a valuable source of vitamin B supplements. The abundance of vitamin B1, B2, B3, and B6 in *S. alterniflora* could be caused by the genectic and climate, weather, and soil condition differences with other food plants.

As for vitamin C content, the *Sa*_Xiapu tender stems had the highest level (1.73 mg/100 g), followed by the *Sa*_Quanzhou (0.88 mg/100 g) and the *Sa*_Lianjiang (0.29 mg/100 g) samples (Figure 3e). However, these levels were lower than those found in celery (3.1 mg/100 g) [57], bamboo shoots (4 mg/100 g) [46], asparagus (5.6 mg/100 g) [56], *Z. latifolia* swollen culms (6 mg/100 g) [49], and leeks (12 mg/100 g) [55].

Overall, the varying vitamin profiles of *S. alterniflora* tender stems highlight their potential as a nutritious dietary supplementation, particularly for those seeking to enhance their intake of essential vitamins. Further research and dietary assessments could allow us to investigate the optimal ways of including *S. alterniflora* tender stems in daily nutrition plans for overall health and wellbeing.

### 3.4. Minerals

The macro- and micro-element contents in the *S. alterniflora* tender stems were measured and are listed in Table 2. The results showed that the *S. alterniflora* tender stems were rich in macro-elements. Notably, due to the halophilic environment of *S. alterniflora*, it was not surprising to find that sodium (Na) was one of the most abundant macro-elements, ranging from 518.52 to 549.62 mg/100 g. This level of Na content was comparable to that found in the ice plant (*Mesembryanthemum crystallinum*) at 545.53 mg/100 g [58], which was significantly higher than in regular shoot or stem vegetables like asparagus (2 mg/100 g) [56], bamboo shoots (4 to 10 mg/100 g) [46,59], *Z. latifolia* swollen culms (6 mg/100 g) [49], celery (80 mg/100 g) [57], and leeks (20 mg/100 g) [55]. Therefore, similar to other seawater-grown vegetables, the tender stems of *S. alterniflora* require soaking and desalting before consumption.

Potassium (K) was also found to be one of the most abundant macro-elements in the tender stems of *S. alterniflora*, ranging from 210.68 to 589.57 mg/100 g. The *Sa*_Lianjiang sample had the highest K content at 589.57 mg/100 g, followed by the *Sa*_Quanzhou (535.77 mg/100 g) and *Sa*_Xiapu (210.68 mg/100 g) samples. This variation could be related to the cultivar and environmental parameter differences among the three regions. When compared with other vegetables, the average K content in the tender stems of *S. alterniflora* (445.34 mg/100 g) was more than twice that in *Z. latifolia* swollen culm (209 mg/100 g) [50], asparagus (202 to 271 mg/100 g) [56,60], and celery (260 mg/100 g) [57] and five times higher than that in bamboo shoots (78 mg/100 g) as reported by USDA [46]. These levels were comparable to, or lower than, certain species of juvenile bamboo shoots reported by Nirmala [59], such as *Bambusoideae giganteus* (288 mg/100 g), *B. tulda* (408 mg/100 g), *B. asper* (464 mg/100 g), and *B. bambos* (576 mg/100 g). The tender stems of *S. alterniflora* contained calcium (Ca) ranging from 27.24 to 41.73 mg/100 g, magnesium (Mg) ranging from 36.55 to 41.62 mg/100 g, and phosphorus (P) ranging from 50.51 to 63.80 mg/100 g. These values were higher than those found in celery [57], asparagus [56], bamboo shoots [46], and *Z. latifolia* swollen culms [51,54]. This suggests that the *S. alterniflora* tender stems are rich in macro-elements, a finding consistent with the ash measurements (Figure 1b).

The zinc (Zn) content in the tender stems of *S. alterniflora* ranged from 479.75 to 506.30 μg/100 g, and the copper (Cu) content ranged from 58.05 to 83.84 μg/100 g. The iron (Fe) content in the tender stems of *S. alterniflora* ranged from 658.16 to 2371.98 μg/100 g, being, on averge, lower than that in asparagus while 1.5 to 9 times higher than those in bamboo and celery [46,57]. The iron content in *Sa*_Lianjiang samples (2371.98 μg/100 g) was more thsn three times than that in *Sa*_Xiapu. The manganese (Mn) content ranged from 606.46 to 1718.38 μg/100 g, which was 2.5 to 8 times higher than those in bamboo shoots and asparagus [46,56]. The selenium (Se) content ranged from 0.8 to 2.01 μg/100 g, which was higher than that in bamboo shoots (0.8 μg/100 g) [46] but lower than that in asparagus (2.3 μg/100 g) [56]. Additionally, we measured the levels of boron (B) and iodine (I) in the tender stems of *S. alterniflora* and found them to be 72.82 to 124.00 μg/100 g and 1.97 to 4.81 μg/100 g, respectively. We observed that the levels of Fe, Mn, and I in *S. alterniflora* from Xiapu, Lianjiang, and Quanzhou varied significantly, being several times higher or lower than those in other regions. This variation is likely associated with coastal environmental factors, such as soil composition, climate and weather changes, and microbial activity, which influence the uptake of these micronutrients.

Overall, the analysis of macro- and micro-elements in the *S. alterniflora* tender stems indicates a significant richness in essential nutrients. These tender stems contain high concentrations of Na, K, Ca, Mg, and P. Notably, the high K levels exceed those found in many common vegetables. The micronutrient composition, including Mn and Se, provides nutritional benefits compared to other conventional vegetables such as asparagus and bamboo shoots. In general, these findings suggest that *S. alterniflora* tender stems are a valuable source of essential macro- and micro-elements.

### 3.5. Total Alkaloids, Total Flavonoids, and Total Phenols

**Figure 4:**
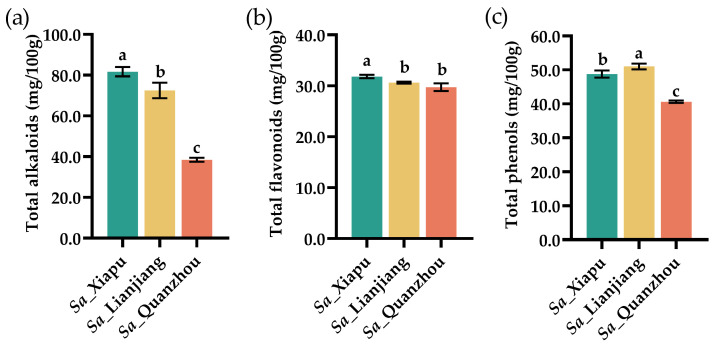
Contents of antioxidants in *S. alterniflora* tender stems (mg/100 g, fresh weight). The amounts of total alkaloids (**a**), total flavonoids (**b**), and total phenols (**c**) were measured in *S. alterniflora* tender stems from the Xiapu (indicated as *Sa*_Xiapu), Lianjiang (*Sa*_Lianjiang), and Quanzhou (*Sa*_Quanzhou) areas. The statistical differences between groups were assessed using *p*-values (<0.05) and represented with alphabetical letters (a–c).

Alkaloids, flavonoids, and phenols are secondary metabolites and have significant roles in health, nutrition, and drug development. Alkaloids are a diverse group of nitrogen-containing compounds found in plants. They often exhibit potent biological activities for medical use, including analgesic, antimalarial, and antitumor effects [61,62]. Flavonoids and phenols are known for their strong antioxidant properties, which help prevent diseases by neutralizing free radicals and reducing inflammation [62]. We also measured the levels of total alkaloids, total flavonoids, and total phenols in the tender stems of *S. alterniflora* (Figure 4).

Among the tested samples, the *Sa*_Xiapu tender stems had the highest levels of total alkaloids at 81.66 mg/100 g (Figure 4a), compared to the *Sa*_Lianjiang (72.45 mg/100 g) and *Sa*_Quanzhou (55.47 mg/100 g) samples. The total alkaloid content in the *S. alterniflora* tender stems was higher than that in *Ephedra alata* (543.79 μg/g), which contains ergot alkaloids [63]. *E. alata* is an important herb in traditional Chinese medicine and has been used for thousands of years. In the 1970s, the fungus *Claviceps purpurea* was isolated from *S. alterniflora* in North America and was found to produce ergot alkaloids [64], which have multiple effects in medical therapy, including the treatment of migraines and applications in labor and delivery. However, an overdose of ergot alkaloids can also lead to adverse effects in humans. Notably, no strains of *C. purpurea* have been reported to be isolated from *S. alterniflora* in China. Future research should focus on the alkaloid compounds in *S. alterniflora* and their potential medical applications.

The *Sa*_Xiapu tender stems also exhibited the highest total flavonoid content at 31.82 mg/100 g (Figure 4b), followed by the *Sa*_Lianjiang (30.62 mg/100 g) and *Sa*_Quanzhou (29.73 mg/100 g) samples. The total flavonoid content in *S. alterniflora* tender stems was lower than that in *E. alata* (38.5 mg/100 g) [63]. The *Sa*_Lianjiang tender stems contained the highest levels of total phenols at 51.02 mg/100 g (Figure 4c), compared to the *Sa*_Xiapu (48.77 mg/100 g) and *Sa*_Quanzhou (40.62 mg/100 g) samples. While this was higher on average than in moso bamboo (*Dendrocalamus latiflorus*) stems (20 to 49 mg/100 g) [65], it was lower than in *Z. latifolia* swollen culms [49] and four species of bamboo shoots, *B. balcooa*, *B. tulda*, *D. giganteus*, and *D. hamiltonii* (191.4 to 505.9 mg/100 g) [66], indicating that *S. alterniflora* could have normal levels of total phenol and flavonoids and could have antioxidant properties.

### 3.6. Antioxidant Activities

**Figure 5:**
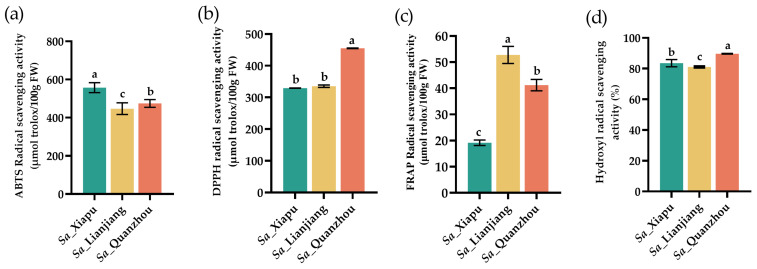
Antioxidant activities of *S. alterniflora* tender stems (fresh weight) were evaluated by ABTS (**a**), DPPH (**b**), FRAP (**c**), and hydroxyl radical scavenging activity (**d**). *Sa*_Xiapu, *Sa*_Lianjiang, and *Sa*_Quanzhou indicate *S. alterniflora* tender stems from the Xiapu, Lianjiang, and Quanzhou areas, respectively. The error bars in each column were calculated based on standard deviation (SD) in three replicates. The statistical differences between groups were assessed using *p*-values (<0.05) and represented with alphabetical letters (a–c).

Flavonoids and phenols have been associated with the antioxidant capacity of plants. Antioxidants can function through various mechanisms. To investigate the potential antioxidant activity in *S. alterniflora*, extracts from its tender stems were evaluated using the ABTS, DPPH, FRAP, and hydroxyl radical scavenging assays (Figure 5).

The ABTS assay is a decolorization method that assesses the ability of antioxidants to neutralize 2,2′-azinobis (3-ethylbenzothiazoline-6-sulfonic acid) (ABTS) radicals. This method is suitable for both water- and lipid-soluble antioxidants, such as flavonoids, hydroxycinnamates, carotenoids, and plasma antioxidants [67]. Among the samples tested, the *Sa*_Xiapu tender stems exhibited the highest ABTS radical scavenging activity (557.19 μmol trolox/100 g), compared to the *Sa*_Lianjiang (447.18 μmol trolox/100 g) and *Sa*_Quanzhou (474.69 μmol trolox/100 g) samples (Figure 5a). This trend aligned with the levels of total alkaloids, flavonoids, and phenols (Figure 4). Compared to *Sa*_Quanzhou, the *Sa*_Xiapu and *Sa*_Lianjiang samples had higher levels of total alkaloids, flavonoids, and phenols, potentially contributing to their stronger ABTS antioxidant activity.

The DPPH assay (2,2-diphenyl-1-picrylhydrazyl) is more effective for lipid-soluble antioxidants, as DPPH radicals are lipid-soluble [68]. We measured the ability of antioxidants in *S. alterniflora* to scavenge DPPH free radicals. The *Sa*_Qaunzhou tender stems had the highest DPPH radical scavenging activity (455.02 μmol trolox/100 g), followed by the *Sa*_Lianjiang (335.422 μmol trolox/100 g) and *Sa*_Xiapu (329.29 μmol trolox/100 g) samples (Figure 5b), suggesting that the *Sa*_Quanzhou sample may contain higher levels of lipid-soluble antioxidants, such as terpenoids, carotenoids, and vitamin E. The ABTS, DPPH radical scavenging activities in the *S. alterniflora* tender stems were higher than those in leek [55], celery [57], and carrot [69] but lower than those in wild asparagus and goji berry fruit [54,70], indicating that *S. alterniflora* contains specific antioxidant compounds with varying levels of activity.

The FRAP assay (ferric reducing antioxidant power) measures the ability of antioxidants to reduce Fe^3+^ to Fe^2+^, which is used to evaluate reducing-type antioxidants. The *Sa*_Lianjiang tender stems had the highest FRAP radical activity (52.75 μmol trolox/100 g), followed by the *Sa*_Quanzhou (41.20 μmol trolox/100 g) and *Sa*_Xiapu (19.16 μmol trolox/100 g) samples (Figure 5c), and this activity level was lower than that in asparagus, leeks, celery, carrots, and goji berry fruit [55,56,57,69,70], indicating a lower accumulation of reducing-type antioxidants, such as glutathione, polyphenols, and vitamin C, in the tender stems of *S. alterniflora*.

Hydroxyl radicals (•OH) are highly reactive and capable of causing significant cellular damage. Antioxidants can neutralize hydroxyl radicals by converting them into less reactive species (e.g., phenolic compounds) or by preventing oxidative chain reactions like lipid peroxidation. In this assay, all tested samples exhibited strong antioxidant activity with notable differences. For example, the Sa_Quanzhou tender stems showed a scavenging rate of 89.67%, followed by those from Sa_Xiapu (83.52%) and from Sa_Lianjiang (81.07%) (Figure 5d). The hydroxyl radical scavenging activity followed a similar pattern to the DPPH radical scavenging activity across the samples. It is possible that specific compounds in *S. alterniflora* contribute to both hydroxyl and DPPH radical scavenging activities.

### 3.7. Food Safety Evaluations

**Figure 6:**
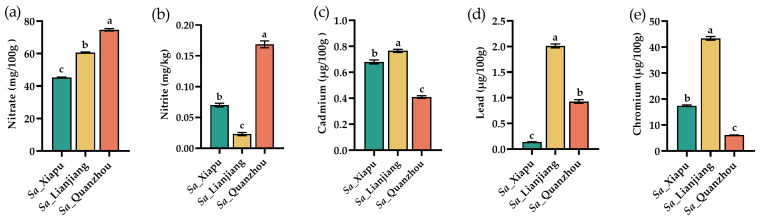
Contents of nitrate (NO_3_^−^), nitrite (NO_2_^−^), and heavy metals (cadmium, lead, chromium) in *S. alterniflora* tender stems (fresh weight). (**a**–**e**) indicates the amount of nitrate (NO_3_^−^), nitrite (NO_2_^−^), cadmium (Cd), lead (Pb), and chromium (Cr) in *S. alterniflora* tender stems, respectively. *Sa*_Xiapu, *Sa*_Lianjiang, *Sa*_Quanzhou indicates *S. alterniflora* tender stems from Xiapu, Lianjiang and Quanzhou areas, respectively. The error bars in each column were determined by calculating the standard deviation (SD) from three replicates. Statistical differences between groups were evaluated using *p*-values (<0.05) and are denoted by alphabetical letters (a–c).

The levels of nitrate (NO_3_^−^) and nitrite (NO_2_^−^) in vegetables are of wide concern due to their toxicity at high levels [71]. The *S. alterniflora* tender stems contained NO_3_^−^ levels ranging from 45.32 to 74.7 mg/100 g (Figure 6a) and NO_2_^−^ levels from 0.18 to 0.73 mg/kg (Figure 6b), all within the maximal limits set by the European Commission (2000–7000 mg/kg for NO_3_^−^, Commission regulation, No 18881/2006) [72] and China (20 mg/kg for NO_2_^−^, GB2762-2022) [73], respectively.

The amounts of heavy metals, including cadmium (Cd), lead (Pb), and chromium (Cr), were evaluated as well in this study. The *S. alterniflora* tender stems contained Cd amounts ranging from 0.67 to 0.89 μg/100 g (Figure 4c) and Pb amounts ranging from 0.137 to 2.01 μg/100 g (Figure 6d). While the concentrations varied across samples, all tested samples met the permissible limits set by both the European Commission (CAC: 0.1 mg/kg for Cd) [72] and China (0.05 mg/kg for Cd, 0.1 mg/kg for Pb) [73]. The total chromium (Cr) amount in the *Sa*_Lianjiang tender stems was determined to be 43.37 μg/100 g, followed by the *Sa*_Xiapu (17.44 μg/100 g) and *Sa*_Quanzhou (6.09 μg/100 g) samples (Figure 6e), with all being within China’s standard limit (0.5 mg/kg or, alternatively, 50 μg/100 g) [73]. Therefore, it is proposed that the consumption of *S. alterniflora* tender stems does not pose a health risk to consumers.

## 4. Conclusions

The *S. alterniflora* tender stems harvested from coastal regions of Fujian, China, have a rich nutritional composition, with higher levels of protein, carbohydrates, and fats. Despite regional variations, the *S. alterniflora* tender stems contain significant amounts of essential vitamins, minerals, and antioxidants, indicating their potential as a nutritious addition to the diet or livestock forage. Contaminants such as nitrate, cadmium, and lead in *S. alterniflora* tender stems are within safe consumption levels. Our results provide valuable information for the comprehensive utilization of *S. alterniflora* resources and will contribute to the integrated control of *S. alterniflora*. In further research, a comparative study over seasons and regions could be carried out to elucidate the mechanism behind this varition.

## Figures and Tables

**Table 1:** Profiles of 20 amino acids in *S. alterniflora* tender stems (mg/100 g, fresh weight).

Amino Acid	*Sa*_Xiapu	*Sa*_Lianjiang	*Sa*_Quanzhou
Essential amino acids			
Histidine (His, H)	10.07 ± 0.57 ^b^	2.8 ± 0.28 ^c^	34.28 ± 2.53 ^a^
Isoleucine (Ile, I)	6.08 ± 0.28 ^a^	4.18 ± 0.23 ^b^	3.36 ± 0.35 ^c^
Leucine (Leu, L)	3.00 ± 0.05 ^b^	1.90 ± 0.08 ^c^	3.81 ± 0.34 ^a^
Lysine (Lys, K)	5.72 ± 0.06 ^a^	1.79 ± 0.18 ^b^	1.53 ± 0.06 ^c^
Methionine (Met, M)	0.66 ± 0.04 ^b^	1.14 ± 0.07 ^a^	1.13 ± 0.04 ^a^
Phenylalanine (Phe, F)	6.97 ± 0.09 ^a^	2.1 ± 0.07 ^b^	2.61 ± 0.16 ^b^
Threonine (Thr, T)	3.31 ± 0.16 ^c^	4.34 ± 0.05 ^a^	3.75 ± 0.11 ^b^
Tryptophan (Trp, W)	5.19 ± 0.39 ^a^	2.19 ± 0.23 ^b^	1.94 ± 0.15 ^c^
Valine (Val, V)	28.61 ± 1.01 ^c^	41.36 ± 1.26 ^a^	39.94 ± 0.7 ^b^
Non-essential amino acids			
Alanine (Ala, A)	11.39 ± 0.32 ^c^	15.23 ± 0.61 ^a^	14.16 ± 0.72 ^a^
Arginine (Arg, R)	6.33 ± 0.34 ^a^	2.11 ± 0.19 ^c^	5.99 ± 0.08 ^b^
Asparagine (Asn, N)	116.45 ± 2.97 ^b^	201.08 ± 3.56 ^a^	61.4 ± 2.34 ^c^
Aspartic acid (Asp, D)	32.91 ± 1.12 ^a^	27.37 ± 1.1 ^b^	10.58 ± 0.61 ^c^
Cysteine (Cys, C)	7.60 ± 0.67 ^a^	4.39 ± 0.15 ^c^	5.27 ± 0.17 ^b^
Glutamic acid (Glu, E)	38.29 ± 3.16 ^a^	31.9 ± 2.32 ^b^	23.34 ± 1.45 ^c^
Glutamine (Gln, Q)	3.94 ± 0.07 ^c^	7.15 ± 0.14 ^b^	10.45 ± 0.07 ^a^
Glycine (Gly, G)	0.58 ± 0.04 ^c^	1.24 ± 0.04 ^b^	2.75 ± 0.18 ^a^
Proline (Pro, P)	38.94 ± 0.78 ^b^	26.67 ± 0.9 ^c^	42.24 ± 2.74 ^a^
Serine (Ser, S)	19.36 ± 1.60 ^b^	26.47 ± 0.43 ^a^	8.81 ± 0.28 ^c^
Tyrosine (Tyr, Y)	11.25 ± 0.82 ^b^	13.34 ± 0.63 ^a^	7.72 ± 0.32 ^c^
TEAA ^1^	69.61 ± 1.49 ^b^	61.79 ± 1.79 ^c^	92.36 ± 1.95 ^a^
TNEAA ^2^	287.04 ± 7.87 ^b^	356.95 ± 6.16 ^a^	192.71 ± 4.29 ^c^
TUAA ^3^	71.21 ± 3.87 ^a^	59.28 ± 1.73 ^b^	33.92 ± 1.07 ^c^
TBAA ^4^	82.71 ± 1.78 ^b^	74.21 ± 2.12 ^c^	90.55 ± 3.26 ^a^
TAA ^5^	356.65 ± 9.17 ^b^	418.73 ± 7.82 ^a^	285.07 ± 2.66 ^c^

^1^ TEAA: total essential amino acid; ^2^ TNEAA: total non-essential amino acid; ^3^ TUAA: total umami amino acid, including glutamic acid and aspartic acid; ^4^ TBAA: total bitter amino acid, including proline, valine, leucine, phenylalanine, and tryptophan; ^5^ TAA: total amino acid. The superscript letters a–c indicate statistical differences (*p* < 0.05).

**Table 2:** Contents of macromineral and micromineral elements in *S. alterniflora* tender stems (fresh weight).

Mineral Elements	*Sa*_Xiapu	*Sa*_Lianjiang	*Sa*_Quanzhou
Macromineral elements (mg/100 g):			
Sodium, Na	549.62 ± 10.08 ^a^	518.52 ± 1.57 ^b^	549.06 ± 2.28 ^a^
Potassium, K	210.68 ± 2.7 ^c^	589.57 ± 3.41 ^a^	535.77 ± 4.52 ^b^
Calcium, Ca	27.24 ± 1.36 ^c^	41.73 ± 1.03 ^a^	38.7 ± 0.38 ^b^
Magnesium, Mg	37.4 ± 0.61 ^b^	41.62 ± 1.09 ^a^	36.55 ± 0.51 ^b^
Phosphorus, P	58.57 ± 1.25 ^b^	50.51 ± 0.19 ^c^	63.8 ± 1.25 ^a^
Trace elements (μg/100 g):			
Iron, Fe	658.16 ± 35.67 ^c^	2371.98 ± 6.65 ^a^	894.42 ± 0.71 ^b^
Manganese, Mn	1247.66 ± 10.08 ^b^	606.46 ± 2.85 ^c^	1718.38 ± 25.79 ^a^
Zinc, Zn	506.19 ± 36.23 ^a^	506.32 ± 2.42 ^a^	479.75 ± 2.11 ^b^
Copper, Cu	83.84 ± 0.39 ^a^	58.05 ± 0.80 ^c^	67.48 ± 0.68 ^b^
Boron, B	124.00 ± 3.55 ^a^	72.82 ± 0.14 ^c^	100.67 ± 2.67 ^b^
Selenium, Se	2.01 ± 0.01 ^a^	1.12 ± 0.03 ^b^	0.8 ± 0.02 ^c^
Iodine, I	4.81 ± 0.05 ^a^	1.97 ± 0.04 ^b^	1.98 ± 0.02 ^b^

Statistical differences between groups were evaluated using *p*-values (<0.05) and are denoted by alphabetical letters (a–c).

## Data Availability

The original contributions presented in the study are included in the article, further inquiries can be directed to the corresponding authors.
